# Dextran-Coated Latex Nanoparticles via Photo-RAFT Mediated Polymerization Induced Self-Assembly

**DOI:** 10.3390/polym13234064

**Published:** 2021-11-23

**Authors:** Valeria Lizeth Romero Castro, Brahim Nomeir, Ana Andreea Arteni, Malika Ouldali, Jean-Luc Six, Khalid Ferji

**Affiliations:** 1 Université de Lorraine, CNRS, LCPM, 54000 Nancy, France; valeromeroC@hotmail.com (V.L.R.C.); brahim.nomeir10@gmail.com (B.N.); jean-luc.six@univ-lorraine.fr (J.-L.S.); 2 Cryo-Electron Microscopy Facility, Institute for Integrative Biology of the Cell (I2BC), Université Paris-Saclay, CEA, CNRS, 91198 Gif-sur-Yvette, France; ana-andreea.arteni@i2bc.paris-saclay.fr (A.A.A.); malika.ouldali@i2bc.paris-saclay.fr (M.O.)

**Keywords:** iniferter, PISA, photo-RAFT polymerization, latex nanoparticle, emulsion, dispersion, graft copolymer, drug delivery, textile, paint

## Abstract

Polysaccharide coated nanoparticles represent a promising class of environmentally friendly latex to replace those stabilized by small toxic molecular surfactants. We report here an in situ formulation of free-surfactant core/shell nanoparticles latex consisting of dextran-based diblock amphiphilic copolymers. The synthesis of copolymers and the immediate latex formulation were performed directly in water using a photo-initiated reversible addition fragmentation chain transfer-mediated polymerization induced self-assembly strategy. A hydrophilic macromolecular chain transfer-bearing photosensitive thiocarbonylthio group (eDexCTA) was first prepared by a modification of the reducing chain end of dextran in two steps: (i) reductive amination by ethylenediamine in the presence of sodium cyanoborohydride, (ii) then introduction of CTA by amidation reaction. Latex nanoparticles were then formulated in situ by chain-extending eDexCTA using 2-hydroxypropyl methacrylate (HPMA) under 365 nm irradiation, leading to amphiphilic dextran-*b*-poly(2-hydroxypropyl methacrylate) diblock copolymers (DH_X_). Solid concentration (SC) and the average degree of polymerization -$\;\overset{ˉ}{X_{n}}$- of PHPMA block (*X*) were varied to investigate their impact on the size and the morphology of latex nanoparticles termed here SCDH_X_. Light scattering and transmission electron microscopy analysis revealed that SCDH_X_ form exclusively spherical nano-objects. However, the size of nano-objects, ranging from 20 nm to 240 nm, increases according to PHPMA block length.

## 1. Introduction

For many decades, latex, consisting of aqueous colloidal polymer-based nanoparticles, has been intensively used in various fields including coatings, textiles, paints, papers, beauty products and nanocarriers for drug delivery [1,2]. Typically, these core/shell nanoparticles are composed of a surfactant that constitutes the hydrophilic corona while the inner core is formed by a hydrophobic polymer. The colloidal stability and the size of these nanoparticles are strongly influenced by the chemical nature and the concentration of the surfactant used in the formulation. Latex nanoparticles are typically prepared in a heterogeneous medium by emulsion polymerization of a water-insoluble monomer stabilized by surfactants of low molecular weight. Despite the efficiency of this process, an excess of surfactants, sometimes toxic to the environment, is necessary to prevent the coalescence of particles. Thus, a great interest in the development of free-surfactant latex was born by using polymeric steric stabilizers [3].

Polysaccharides are a broad class of non-toxic natural polymers. The broad diversity of functional groups, such as amine and hydroxyl, present on their backbone offers to polysaccharide derivatives strong adsorption behavior in interfaces leading them to be attractive eco-friendly stabilizers for latex synthesis [4,5,6,7,8,9]. Native polysaccharides [10] or hydrophobically modified polysaccharides [11,12,13] have already been used to sterically stabilize monomer droplets during emulsion or mini-emulsion polymerization. However, the main limitation of this strategy is the potential desorption of polysaccharides in a harsh medium [14,15,16]. To prevent such phenomenon, polysaccharides were covalently linked to the hydrophobic core in situ using click chemistry [15] or by using pre-synthesized polysaccharide-based amphiphilic copolymers that form nanoparticles in water [17,18,19,20]. However, the multiple processing and purification steps required for the synthesis and purification of such copolymers and the low solid concentration (typically < 1% *w*/*w*) of nanoparticles produced, are the main disadvantages of these strategies [21,22].

To overcome these limitations, an environmentally friendly process has been developed to produce stable nanoparticles of a covalently linked corona to core at a high solid concentration (up to 40% wt) [23,24,25]. Such technology, known as aqueous polymerization induced self-assembly (PISA) [26,27,28,29,30,31,32], involves a chain-extension of a hydrophilic macromolecular stabilizer using a monomer that forms a hydrophobic block when reaching a critical chain-length. Subsequently, amphiphilic copolymers are formed and self-assembled in situ into nano-objects. As we demonstrated in our recent mini-review [33], the ‘one-pot synthesis and self-assembly’ concept of PISA technology is particularly interesting for polysaccharides-containing amphiphilic copolymers to overcome the limitation of their self-assembly using the multi-steps solvent-switch methods [33]. Indeed, these methods require a common organic solvent for both parts of the preformed copolymer, hydrophilic polysaccharide and hydrophobic synthetic polymer, which is rarely fulfilled. Recently, the scope of PISA has been extended to natural polysaccharides used as steric stabilizers, including dextran [21,32,34,35], alginate [36], xyloglucane [37], and mannuronan/guluronan [38]. Among these systems, graft copolymers associating dextran as hydrophilic backbone and poly(2-hydroxypropyl methacrylate) (PHPMA) as hydrophobic grafts is the only system showing formation of nano-objects of advanced morphologies, such as worm-like micelles and vesicles [21,32,34]. It should be noted that reports on nano-objects bearing sugar moieties such as glucose [39] or galactose [40] using PISA are not considered as they are out of the scope of the current investigation.

Herein, we varied the architecture of our previous system [21,32,34], from graft to diblock, to investigate the impact of the polysaccharide-based copolymers architecture in PISA. For that purpose, an end-functionalized dextran bearing a photo-sensitive thiocarbonylthio chain transfer RAFT agent (eDexCTA) was prepared, then chain-extended using 2-hydroxypropyl methacrylate (HPMA) in water to produce nano-objects of dextran-*b*-PHPMA diblock copolymers. The final copolymer concentration (SC) in the medium and the average degree of polymerization -$\;\overset{ˉ}{X_{n}}$- of PHPMA block (*X*) were varied to investigate their impact on the assembly. In the following paragraphs, produced nano-objects suspensions will be called SCDH_X_; SC is the targeted solid concentration, D is dextran, H is PHPMA, and X is the average degree of polymerization -$\;\overset{ˉ}{X_{n}}$- of PHPMA block. Characterization of the obtained nano-objects by dynamic and static light scattering (DLS, SLS) and by transmission electron microscopy (TEM) revealed that all SCDH_X_ copolymers form latex nano-objects of spherical morphology. The size of the nano-objects varies depending on the experimental conditions studied.

## 2. Materials and Methods

### 2.1. Chemical Reagents

**Scheme 1:**
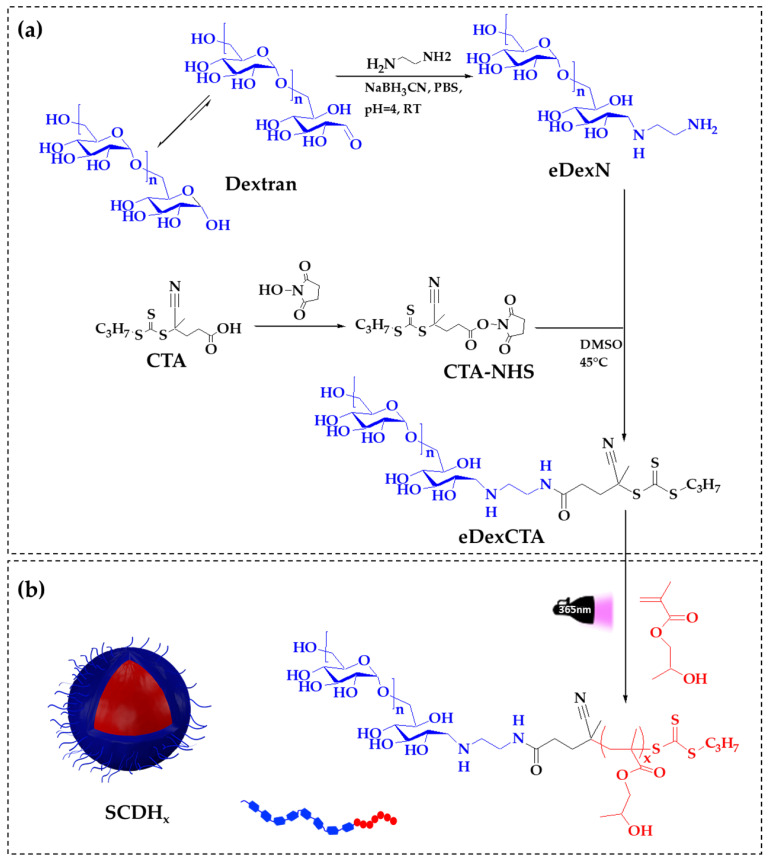
Synthetic pathway of (**a**) the macromolecular chain transfer agent (eDexCTA) by end-chain functionalization of dextran (D) with CTA, and (**b**) SCDH_x_ nano-objects by aqueous photo-PISA of HPMA from eDexCTA at room temperature. *X* is the average degree of polymerization -$\;\overset{ˉ}{X_{n}}$- of PHPMA block (H), and SC is the solid concentration.

Dextran (${\overset{ˉ}{M}}_{n}$ = 4760 g/mol, Ð = 1.34 determined using SEC in H_2_O/NaNO_3_ (8.5 g/L)), ethylenediamine (>99.5%) and sodium cyanoborohydride (95%) were purchased from Sigma Aldrich. 2-hydroxypropyl methacrylate (HPMA) (98%), containing 25 mol% of 2-hydroxyisopropyl methacrylate (HIPMA) isomer, was purchased from ABCR and passed through a silica gel column before use to remove inhibitors. In this study, only the chemical structure of HPMA will be considered to simplify the chemical structure of the polymer produced. 4-(propylthiocarbonothioylthio)-4-cyanopentanoic acid (CTA) and the activation of its carboxylic acid group by N-hydroxysuccinimide (NHS) to form CTA-NHS were carried out as we reported previously (Scheme 1) [22].

### 2.2. Instruments

#### 2.2.1. NMR Spectroscopy

End-functionalization of dextran and monomer conversion were determined in DMSO-d_6_ using a Brüker Advance 300 MHz NMR spectrometer.

#### 2.2.2. Size Exclusion Chromatography (SEC)

The number-average molar mass (${\overset{ˉ}{M}}_{n}$) and the dispersity (Ð) of native dextran and end-functionalized dextran were determined by SEC in H_2_O/NaNO_3_ (8.5 g/L) at room temperature at 0.7 mL/min using a refractive index increment (dn/dc = 0.147). Experiments were performed using an HPLC pump (waters 515), a degasser AF (Degasys DG-1310), three PL aquagel-OH (40 and 50) 8 μm, a multi-angle laser light scattering (MALLS) detector (Wyatt Technology- Toulouse France, Mini Dawn, with a He–Ne light wavelength at 632.8 nm), and a differential refractometer detector (RID 10A, Shimadzu).

#### 2.2.3. Dynamic/Static Light Scattering (DLS/SLS)

Light-scattering measurements were performed using an ALV/CGS-3 compact goniometer system, equipped with a multiple tau digital correlator (ALV-7004) and a vertically polarized He-Ne laser (22 mW output power) operating at *λ* = 632.8 nm at 20 °C. Scattered light was detected at scattering angles 20° ≤ θ ≤ 150°, corresponding to scattering vector regime 0.00459 nm^−1^ ≤ *q* ≤ 0.00255 nm^−1^. *q* is calculated using Equation (1), where *n* is the refractive index of the solvent (water).

(1) $$q=\frac{4\pi n}{\lambda }\sin(\frac{\theta }{2})$$

In DLS, the autocorrelation functions were analyzed in terms of relaxation time (τ) distribution according to the REPES routine. Z-average hydrodynamic radius (*R_H_*) was estimated using the Stokes–Einstein Equation (2), where *D*_0_ is the diffusion coefficient of the nano-objects determined from the slope of the *q*^2^ dependence of relaxation rate (<Γ> = *Dq*^2^), *k_B_* is the Boltzmann constant, *T* is the absolute temperature and *η_s_* is the viscosity of the solvent (water). The polydispersity (PDI) was determined at *θ* = 90° using the second-order cumulant analysis.

(2) $$R_{H}=\frac{k_{B}T}{6\pi \eta_{s}D_{0}}$$

In SLS, the $\frac{Kc}{R_{\theta }}$ ratio was recorded to determine the radius of gyration (*R_g_*) using the Zimm Equation (3), where *R_θ_* is the average scattered intensity measured at angle *θ*. *K* is an optical constant, *c* is the concentration of the dispersion, A_2_ is the second virial coefficient of the dispersion and $\overset{ˉ}{M_{w}^{nano}}$ is the weight-average molar mass of the nano-objects. *K* is calculated using Equation (4), where *N_A_* is Avogadro’s constant, and dn/dc is the refractive index increment of the nanostructure dispersion (*dn/dc* = 0.145 mL·g^−1^), measured in water using a differential refractometer Waters 410.

(3) $$\frac{Kc}{R_{\theta }}=\frac{1}{\overset{ˉ}{M_{w}^{nano}}P\left(\theta \right)}+2A_{2}c=\frac{1}{\overset{ˉ}{M_{w}^{nano}}}\;\left(1+\frac{1}{3}R_{g}^{2}q^{\;2}\right)+2A_{2}c\;$$

(4) $$K=\frac{4\pi^{2}n^{2}}{N_{A}\lambda^{4}}{\left(\frac{dn}{dc}\right)}^{2}$$

The term 2A_2_c in Equation (3) can be overlooked for diluted systems. Thus, a form factor *P*(*θ*)*_exp_* of the nano-objects can be calculated at each angle (*θ*) using Equation (5). A normalized form factor (6) is calculated relative to the form factor at *θ* = 20° to analyze SLS data.

(5) $$P{\left(\theta \right)}_{exp}=\frac{R_{\theta }}{Kc}\times \frac{1}{\overset{ˉ}{M_{w}^{nano}}}$$

(6) $$\frac{P{\left(\theta \right)}_{exp}}{P{\left(\theta =20{}^{\circ}\right)}_{exp}}=\frac{\frac{Kc}{R\left(\theta =20{}^{\circ}\right)}}{\frac{Kc}{R\left(\theta \right)}}$$

#### 2.2.4. Transmission Electron Microscopy (TEM)

Samples were analyzed by conventional electron microscopy using the negative staining method. Three microliters of the nano-objects dispersion (1 mg/mL) were deposited on an air glow-discharged carbon-coated grid for 1 min. The excess of liquid was blotted, and the grid rinsed with 2% *w*/*v* aqueous uranyl acetate. The grids were visualized at 100 kV with a Tecnai 12 Spirit transmission electron microscope (Thermo Fisher, New York, NY, USA) equipped with a K2 Base 4 k × 4 k camera (Gatan, Pleasanton, CA, USA). Magnification was at 4400, 6500 or 15,000×, corresponding to a pixel size at the level of the specimen of 0.83, 0.55 and 0.25 nm, respectively.

### 2.3. Synthesis

#### 2.3.1. Preparation of Macromolecular Chain Transfer Agent (eDexCTA)

Synthesis of dextran end-bearing CTA group (eDexCTA) was carried out in two-steps (Scheme 1). The first step involved the functionalization of the end-dextran chain by ethylenediamine to prepare end-aminated dextran (eDexN). The second step involved the functionalization of eDexN by CTA-NHS via an amidation reaction.

Step-1: end-functionalization of dextran by ethylenediamine (synthesis of eDexN):

Ethylenediamine (1.08 mL, 16 mmol) was added to a solution of dextran (5 g, 1.07 mmol) in 50 mL of water at pH = 4 and stirred during 48 h at room temperature. NaBH_3_(CN) (330 mg, 5.4 mmol) was then added, and the reaction mixture was continuously stirred for additional 48 h. After neutralization of the medium by NaOH to reach pH = 7, the mixture was purified by a partial evaporation of water under reduce pressure at 50 °C, followed by precipitation into methanol. eDexN was collected by filtration and dried overnight under vacuum. A white powder was then obtained and characterized by NMR in DMSO-d_6_ and by SEC-MALLS in water/NaNO_3_.

Step-2: end-functionalization of eDexN by CTA-NHS (synthesis of eDexCTA):

eDexN (4.25 g, 0.9 mmol) and CTA-NHS (4.6 g, 4.6 mmol) were dissolved in 50 mL of DMSO. After 36 h of stirring at 45 °C, the dark orange mixture was precipitated twice into methanol and eDexCTA was recovered by filtration and dried overnight under vacuum at 40 °C, then characterized by ^1^H NMR in DMSO-d_6_ and by SEC-MALLS in water/NaNO_3_ (8.5 g/L).

**Figure 1:**
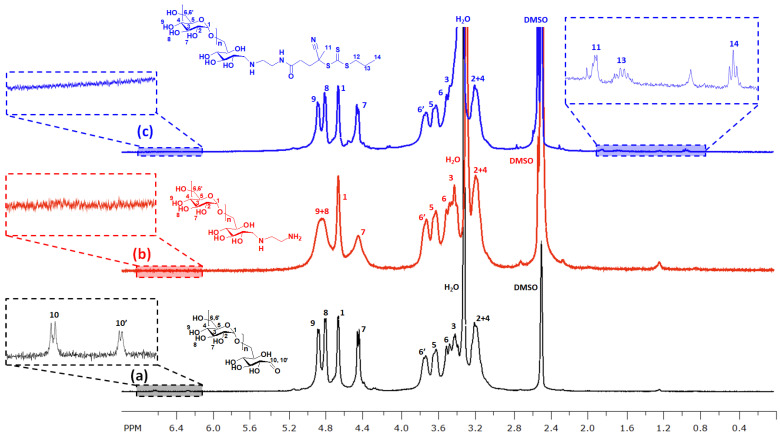
^1^H NMR spectra in DMSO-d_6_ of (**a**) native dextran, (**b**) eDexN, and (**c**) eDexCTA.

The yield of functionalization (*y*) was determined by ^1^H NMR (Figure 1), using Equation (7) where *A*_(1+7+8+9)_ is the integration of the hydroxyl functions (OH_7, 8, 9_) and anomeric protons (H_1_) of the dextran backbone (δ = 4.2–5.0 ppm, 4 n H with *n* = 30 is the number-average of glucopyranosic units of dextran). *A*_(14)_ is the integration of the methyl protons (H_14_) of the CTA group (δ = 0.8 ppm, 3 H).

(7) $$y=\frac{\frac{A_{\left(14\right)}}{3}\;}{\frac{A_{\left(1+7+8+9\right)}}{4\times 30}\;}\times 100$$

#### 2.3.2. Latex Formulation via Photo-RAFT PISA

The solid concentration is calculated by dividing the mass of HPMA (*m_HPMA_*) and of eDexCTA (*m_eDexCTA_*) per the mass of all reagents (HPMA, eDexCTA and water) using the Equation (8):

(8) $$solid\;concentration\;\left(\%\right)=\frac{m_{HPMA}+m_{eDexCTA}}{m_{HPMA}+m_{eDexCTA}+m_{water}}\times 100$$

In a typical reaction for the synthesis of 5%DH_100_ ($\overset{ˉ}{X_{n}}$ = 100 for the PHPMA block at 5% solid concentration): eDexCTA (67 mg, 0.0098 mmol of CTA groups) and HPMA (132 μL, 0.98 mmol) were dissolved in 3.94 mL of milli-Q. The solution was purged with nitrogen for 15 min, then irradiated under stirring for 20 h with a UV lamp (365 nm, light intensity of 4 mW/cm^−2^) to achieve a full conversion (Figures S1–S4).

## 3. Results and Discussion

### 3.1. Synthesis of the Macromolecular Chain Transfer Agent (eDexCTA)

In our previous works [21,32,34], nano-objects of dextran-based graft copolymers (dex-g-PHPMA) were produced using photo-RAFT-mediated PISA carried out from a multi-functionalized dextran based macromolecular chain agent (DexCTA) as a polymeric steric stabilizer. Such a DexCTA was obtained by grafting a photo-sensitive thiocarbonylthio type CTA onto the dextran backbone via an amide bond. [22] In the present report, we target nano-objects based on block copolymer containing dextran to study the effect of the copolymer architecture on the nano-objects morphology in PISA. For this purpose, the CTA was selectively linked to the end of dextran in two steps (Scheme 1).

First, an amine group was introduced at the reducing end of dextran by reductive amination with ethylenediamine in water at pH = 4.0. Due to the presence of two amine functions in ethylenediamine, the excess of this latter was used to prevent a probable coupling of two dextran chains. Sodium cyanoborohydrate was then added to reduce the imine to amine group. The end-aminated dextran (eDexN) was then recovered by precipitation into methanol. ^1^H NMR spectrum (Figure 1b) of eDexN shows a full disappearance of peaks characteristics of the anomeric proton of the terminal glucopyranosic unit of dextran (10 and 10′, Figure 1a), indicating successful end-functionalization of dextran with ethylenediamine. It should be noticed that the peaks of methylene group of ethylenediamine are not visible in the ^1^H NMR spectrum, because they are overlapped with protons of dextran in the region 2.5–4 ppm.

**Figure 2:**
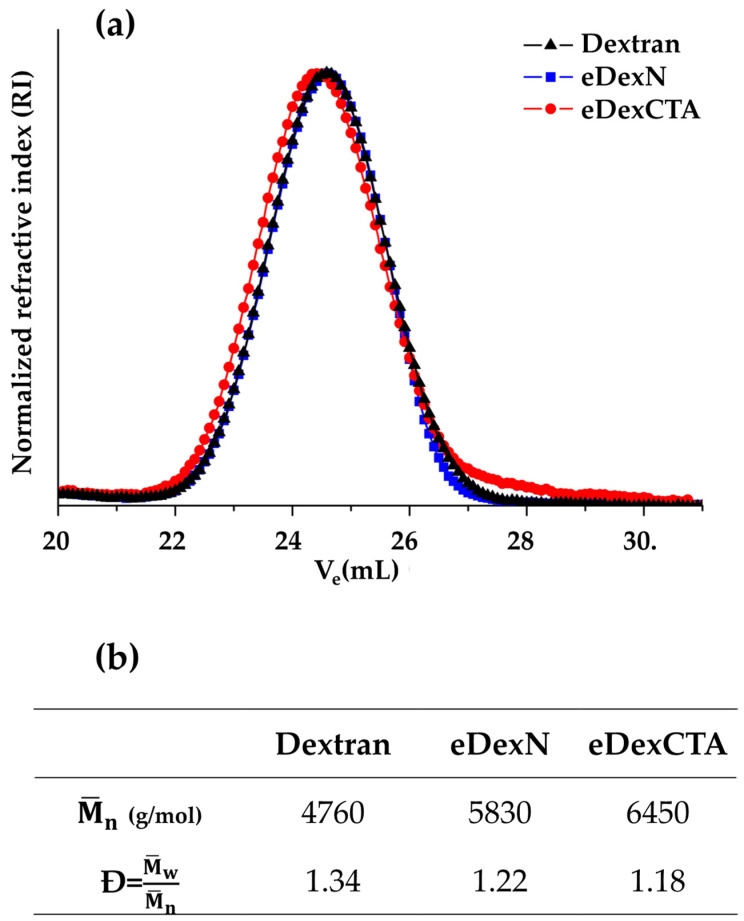
(**a**) SEC traces and (**b**) macromolecular parameters of native dextran, eDexN and eDexCTA in H_2_O/NaNO_3_ at room temperature.

In the second step, the end-functionalization of eDexN by CTA group was performed by mixing the activated acid function of CTA (NHS-CTA) with the eDexN during 36 h at 45 °C. ^1^H NMR spectrum (Figure 1c) of purified eDexCTA shows the appearance of different proton peaks of the CTA group in the region of 0.8–2 ppm, confirming successful end-functionalization of dextran with CTA. A good average coupling efficiency (70%) can be quantified from ^1^HNMR spectrum using Equation (7), equivalent to the efficiency usually reported in the literature for end-functionalization of dextran [41,42]. Figure 2 shows SEC traces of native dextran, eDexN and eDexCTA. A small shift could be observed by comparing traces of eDexCTA with those of dextran and eDexN, which could be attributed to the difference of their solvation in H_2_O/NaNO_3_ and consequently of their hydrodynamic radii. In addition, the significant increase in molar masses of eDexN (${\overset{ˉ}{M}}_{n}$ = 5830 g·mol^−1^) and eDexCTA (${\overset{ˉ}{M}}_{n}$ = 6450 g.mol^−1^) comparing to native dextran (${\overset{ˉ}{M}}_{n}$ = 4760 g·mol^−1^) is attributed to the value of *dn*/*dc* = 0.147 determined for native dextran, which should be different to the values of eDexN and eDexCTA. The key information here is that the experimental conditions do not degrade the § dextran backbone.

### 3.2. Latex Formulation via Photo-RAFT PISA

In PISA, RAFT polymerization is by far the most polymerization technique used for the hydrophobic polymer growth from the hydrophilic polymer steric stabilizer [43,44,45], thanks to its simple experimental setup and its tolerance to a wide range of solvents and chemical functions [46,47,48,49,50]. Here, due to the photo-sensitive behavior of CTA groups present on the eDexCTA, which play a dual role of chain transfer and initiator, RAFT polymerization was performed under UV irradiation (λ = 365 nm) without adding external initiator [32]. As shown in Table 1, different degree of polymerization -$\;\overset{ˉ}{X_{n}}$- of PHPMA block (X = 50, 100, 200, and 300) and various final copolymers concentration at full monomer conversion (SC = 5, 7.5, 10, and 12.5% *w*/*w*) were targeted to produce suspensions of nano-objects based on dextran-*b*-PHPMA diblock copolymers, termed here SCDH_X_. After 20 h of UV irradiation, complete monomer conversions were achieved for almost all suspensions as summarized in Table 1 and Figures S1–S4. Note that copolymers were not characterized by SEC due to the insolubility of dried copolymers powder in DMSO or any other common organic solvent, unlike dextran-*g*-PHPMA graft copolymers. So far, we have no explanation for the phenomenon.

Concentrated suspensions of nano-objects prepared at different solid concentrations were diluted and analyzed by light scattering technique. Dynamic light scattering (DLS) was used to determine the Z-average hydrodynamic radius (*R_H_*) of nano-objects. Static light scattering (SLS) was used to estimate their radius of gyration (*R_g_*) when the size of nano-ojects is large enough to observe an angular dependence of light scattering (at least 40 nm). The ratio *ρ* = *R_g_*/*R_H_* was calculated to obtain a primary indication of the morphology of scattering nano-objects. Table 1 summarizes the characteristics of formulated nano-objects. First, we studied PISA by setting the solid concentration and varying the size of the PHPMA block (X = 50 to 300). For PISA made at 7.5, 10 and 12.5%, DLS shows that size of nano-objects increases with X. For instance, at 12.5% *R_H_* increases from 22 nm (PDI = 0.22) for X = 50 (12.5%DH_50_) to 175 nm (PDI = 0.05) for X = 300 (12.5%DH_300_). Nano-objects formed at 5% with small PHPMA blocks (X = 50) are larger than those formed with X = 100. This can be explained by the high dispersity of 5%DH_50_ (see Figure S5).

SLS also shows that *R_g_* increases by extending the size of the PHPMA block at a fixed concentration. As shown in Table 1, *ρ* is close to 0.77 for most of the suspensions, suggesting that nano-objects have a spherical morphology. To get additional information of nano-objects morphology, the normalized light scattering form factor ($\frac{P{\left(\theta \right)}_{\exp}}{P{\left(\theta =20{}^{\circ}\right)}_{\exp}}$) determined by SLS analysis was fitted by a theoretical expression ($\frac{P{\left(\theta \right)}_{\mathrm{the}}}{P{\left(\theta =20{}^{\circ}\right)}_{\mathrm{the}}}$) using a form factor of sphere of radius (R_fit_) calculated by the Equation (9):

(9) $$P_{2}\left(\theta \right)=\frac{9}{{\left({\mathrm{qR}}_{\mathrm{fit}}\right)}^{6}}\left(\sin\left({\mathrm{qR}}_{\mathrm{fit}}\right)-{\mathrm{qR}}_{\mathrm{fit}}\cos\left({\mathrm{qR}}_{\mathrm{fit}}\right)\right)2$$

**Figure 3:**
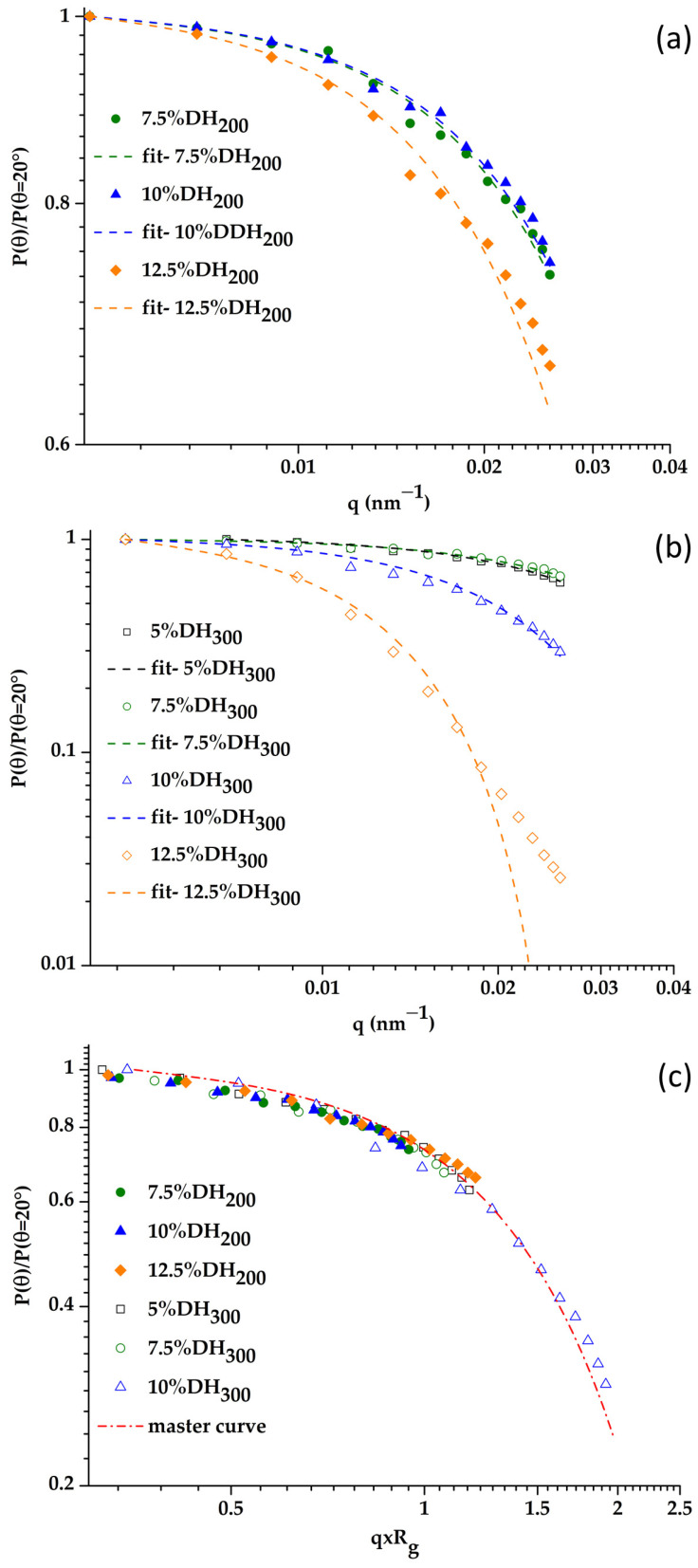
Dependence of experimental normalized form factors ($\frac{P{\left(\theta \right)}_{\exp}}{P{\left(\theta =20{}^{\circ}\right)}_{\exp}}$) and the corresponding fit on (**a**,**b**) scattering vector (q) and (**c**) qxR_g_ of dextran-based diblock copolymers nano-objects prepared by photo-PISA.

The dependence of normalized form factors ($\frac{P{\left(\theta \right)}_{\exp}}{P{\left(\theta =20{}^{\circ}\right)}_{\exp}}$) on scattering vector (q) and the corresponding fit of each suspension are shown in a double-logarithmic scale in Figure 3a,b. Values of spheres radii (R_fit_) used in the Equation (9) are summarized in Table 1. The experimental curves could quantitatively be well described by the Equation (9) for all suspensions. More important, a universal master curve is obtained when plotting the dependance of $\frac{P{\left(\theta \right)}_{\exp}}{P{\left(\theta =20{}^{\circ}\right)}_{\exp}}$ on qxR_g_ (Figure 3c), indicating that all nano-objects exhibit a similar morphology (sphere) independently on the nanoobject molar mass [51]. Thus, we assume that SCDH_X_ dextran-based diblock copolymers exclusively form spherical latex nano-objects in water. Note that the small deviation observed in case of 12.5%DH_300_ at large q is probably due to the polydispersity of nano-objects (Figure 3b).

**Figure 4:**
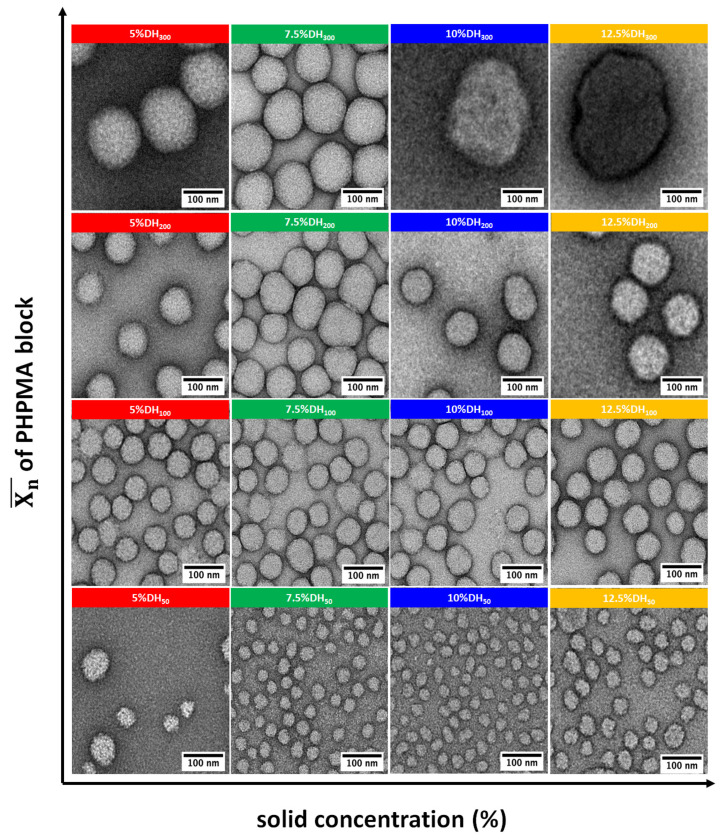
TEM images of SCDH_X_ suspensions formulated with dextran-*b*-PHPMA of different PHPMA size (X = 50, 100, 200, and 300), prepared via photo-RAFT PISA at different solid concentration (5, 7.5, 10 and 12.5% *w*/*w*).

The spherical morphology of the nano-objects formed by PISA was verified using transmission electron microscopy (TEM). To this end, all suspensions were diluted to 1 g/L then observed. The TEM images summarized in Figure 4 confirms our hypothesis. Indeed, spheres of nanometric size (ranging from 20 nm to 240 nm) were observed in all the experimental conditions used. In addition, at a fixed concentration, one can notice that the size of nano-objects increases according to the length of PHPMA block, in agreement with our previous observation using light scattering (Table 1). However, the concentration does not have a significant impact. At a fixed PHPMA block length, nano-objects exhibit a size of close magnitude. Additional images and statical analysis are available in Figures S5–S8 in the supporting information.

It is well known that the concentration and the size of the hydrophobic block are the keys parameters influencing the morphology of nano-objects in PISA. This has been widely reported in case of PHPMA core forming nano-objects, which can form easily large set of nano-objects morphologies [52,53,54,55,56,57,58]. However, in case of the present investigation, we observed exclusively spherical micelles. This limited morphology can be attributed to the diblock architecture of the current copolymer since the graft architecture of copolymer-based on dextran as hydrophilic stabilizer and on PHPMA as hydrophobic grafts, previously reported by us, forms various morphologies such as worm-like micelles and vesicles [21,32,34]. To the best of our knowledge, the present work reveals for the first time that the morphology of nano-objects in PISA depends also on the architecture of the amphiphilic copolymers targeted.

## 4. Conclusions

Dextran-*b*-PHPMA diblock copolymers-based nano-objects were formed for the first time by photo-RAFT mediated PISA in dispersion conditions. First, a macromolecular chain transfer based on dextran (eDexCTA) was prepared in two steps. The dextran chain was extended by polymerizing HPMA in water to form diblock copolymers. Light scattering and TEM analysis revealed formation of latex nano-objects with spherical morphology. The size of the spheres could be tuned by varying the PHPMA length. The exclusive formulation of spherical morphology with dextran-based diblock copolymer is surprising since the equivalent copolymer of graft architecture forms diverse morphologies [21,32,34]. The present work is a demonstration that, in PISA, the architecture of amphiphilic copolymers can have a significant impact on the morphology of nano-objects formed.

## Figures and Tables

**Table 1:** Summary of the characteristics and parameters used to fit the SLS data of different dextran-based nano-objects prepared via photo-RAFT PISA.

Suspension (SCDH_X_)	SC	X	Conv.(%) ^a^	R_TEM_ (nm) ^b^	R_H_ (nm) ^c^	PDI ^d^	R_g_ (nm) ^e^	R_g_/R_H_	R_fit_(nm)
5%DH_50_	5%	50	90	28 ± 14	38	0.20	-	-	-
5%DH_100_	5%	100	>99	31 ± 4	33	0.19	-	-	-
5%DH_200_	5%	200	98	37 ± 5	36	0.12	-	-	-
5%DH_300_	5%	300	98	80 ± 24	64	0.07	46	0.72	60
7.5%DH_50_	7.5%	50	>99	17 ± 2	18	0.50	-	-	-
7.5%DH_100_	7.5%	100	98	29 ± 4	31	0.24	-	-	-
7.5%DH_200_	7.5%	200	>99	40 ± 7	43	0.08	37	0.86	49
7.5%DH_300_	7.5%	300	94	51 ± 6	54	0.01	42	0.77	55
10%DH_50_	10%	50	>99	16 ± 2	19	0.48	-	-	-
10%DH_100_	10%	100	>99	30 ± 4	32	0.24	-	-	-
10%DH_200_	10%	200	>99	39 ± 6	47	0.11	36	0.77	48
10%DH_300_	10%	300	97	107 ± 18	99	0.01	75	0.76	95
12.5%DH_50_	12.5%	50	>99	21 ± 3	22	0.22	-	-	-
12.5%DH_100_	12.5%	100	>99	34 ± 6	32	0.08	-	-	-
12.5%DH_200_	12.5%	200	>99	59 ± 10	76	0.10	44	0.58	60
12.5%DH_300_	12.5%	300	97	238 ± 72	175	0.05	134	0.77	175

^a^ monomer conversion calculated by ^1^H NMR of crude PISA suspensions after 20 h of irradiation at 365 nm (Figures S1–S4). ^b^ average radius measured for ≥100 nano-objects using ImageJ software (Figures S5–S8). ^c^ hydrodynamic radius obtained from multi-angle DLS analysis. ^d^ polydispersity index determined by DLS analysis at *θ* = 90° using the second-order cumulant analysis. ^e^ radius of gyration obtained from SLS analysis using zimm formalism.

## Data Availability

Data presented in this study are available upon request from the corresponding author.

## Supplementary Materials

The following are available online at https://www.mdpi.com/article/10.3390/polym13234064/s1: Figures S1–S4: ^1^H NMR spectra in DMSO-d_6_ of crude suspensions of dextran-*b*-PHPMA_X = 50–300_ prepared via photo-RAFT PISA at 5, 7.5, 10, and 12.5% solid concentration, respectively. Figures S5–S8: TEM images of dextran-*b*-PHPMA_X=50–300_ prepared via photo-RAFT PISA at 5, 7.5, 10, and 12.5% solid concentration. The panels on the right are the statistical analysis of the radii measured for 50 nano-objects using ImageJ software.
