# Supplementary material for: Dextran-Coated Latex Nanoparticles via Photo-RAFT Mediated Polymerization Induced Self-Assembly
Source: Polymers (Basel). 2021 Nov 23;13(23):4064. doi: 10.3390/polym13234064 (PMC8658814; doi:10.3390/polym13234064)
Supplement: Supplementary file 1 [file polymers-13-04064-s001.zip › polymers-1476009-supplementary.pdf]

Supporting Information.

Dextran Coated Latex Nanoparticles *via* Photo-RAFT mediated Polymerization Induced Self-Assembly

Valeria Lizeth Romero Castro[1], Brahim Nomeir[1], Ana Andreea Arteni[2], Malika Ouldali[2], Jean-Luc Six,[1] And Khalid Ferji[1,*]

µ[1] Université de Lorraine, CNRS, LCPM, F-54000 Nancy, France 1 ; khalid.ferji@univ-lorraine.fr; jean-luc.six@univ-lorraine.fr; valeromeroC@hotmail.com; brahim.nomeir10@gmail.com.

[2] Institute for Integrative Biology of the Cell (I2BC), Cryo-electron Microscopy Facility, Université Paris-Saclay, CEA, CNRS; ana-andreea.arteni@i2bc.paris-saclay.fr; malika.ouldali@i2bc.paris-saclay.fr.

* Correspondence: khalid.ferji@univ-lorraine.fr; Tel.: +33-(0)372743691

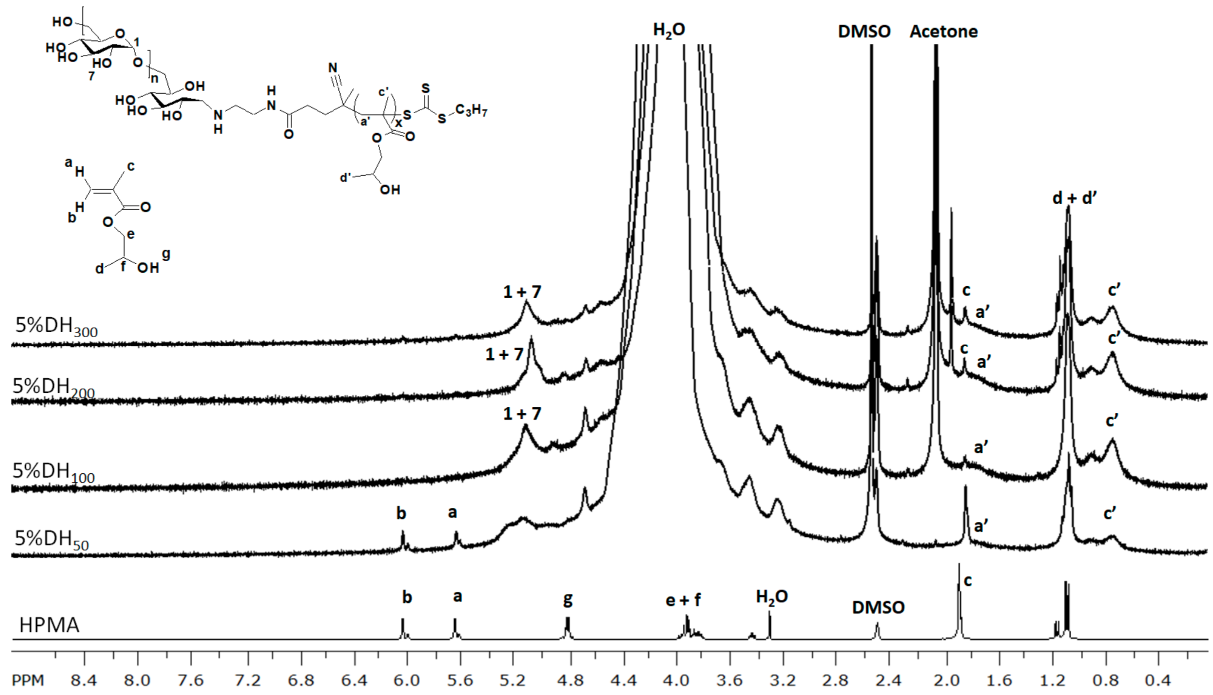

Figure S1. ^{1}H NMR spectra in DMSO-d6 of HPMA and crude suspensions of dextran-*b*-PHPMA$_{X=50-300}$ prepared via photo-RAFT PISA at 5% solid concentration.

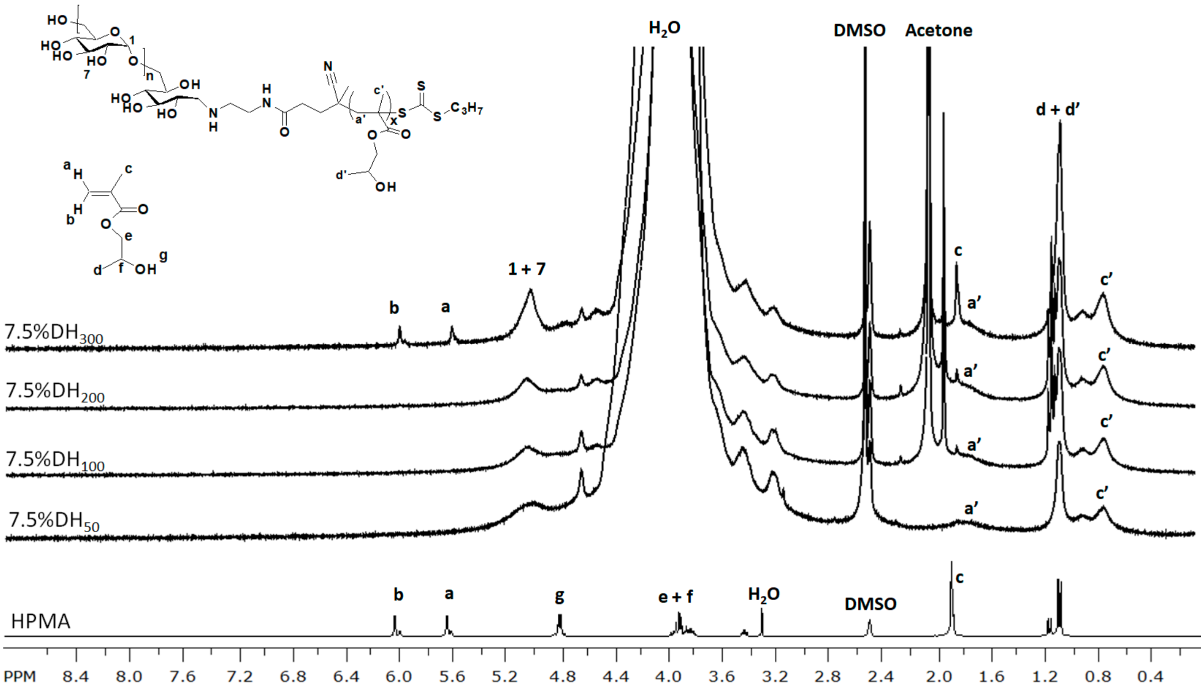

Figure S2. ^1H NMR spectra in DMSO-d$_6$ of HPMA and crude suspensions of dextran-*b*-PHPMA$_{X=50\text{-}300}$ prepared via photo-RAFT PISA at 7.5% solid concentration.

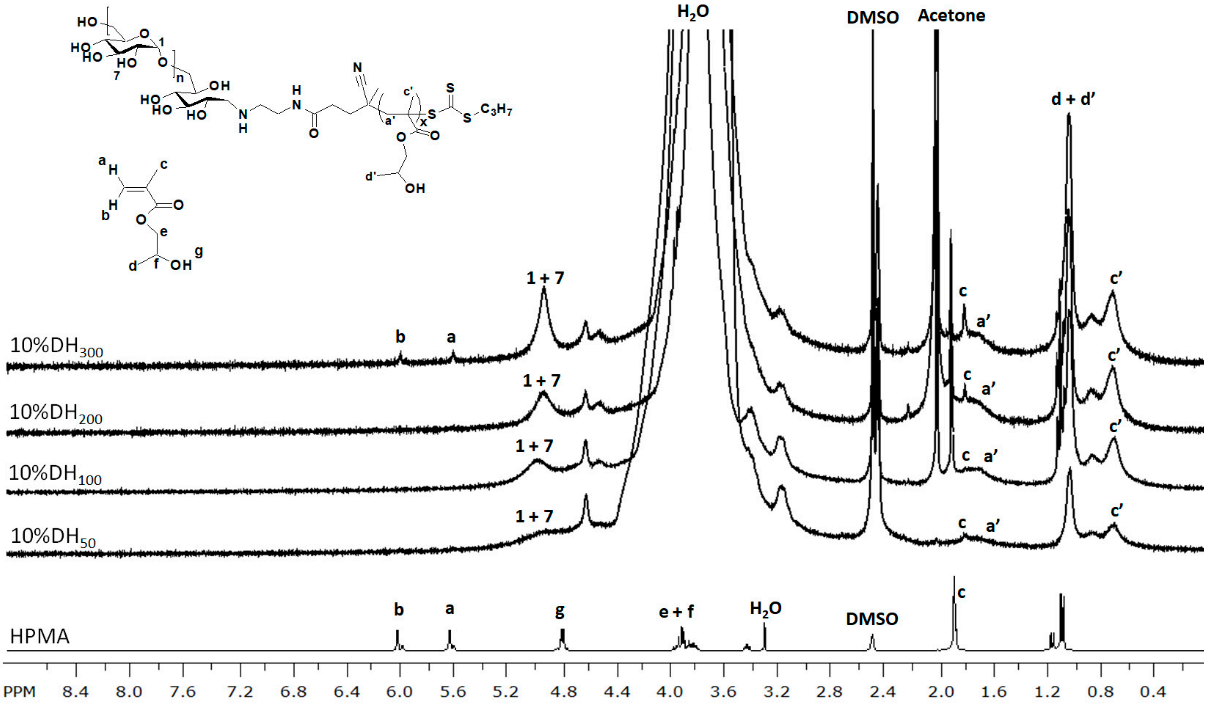

Figure S3. ^1H NMR spectra in DMSO-d$_6$ of HPMA and crude suspensions of dextran-*b*-PHPMA$_{X=50\text{-}300}$ prepared via photo-RAFT PISA at 10% solid concentration.

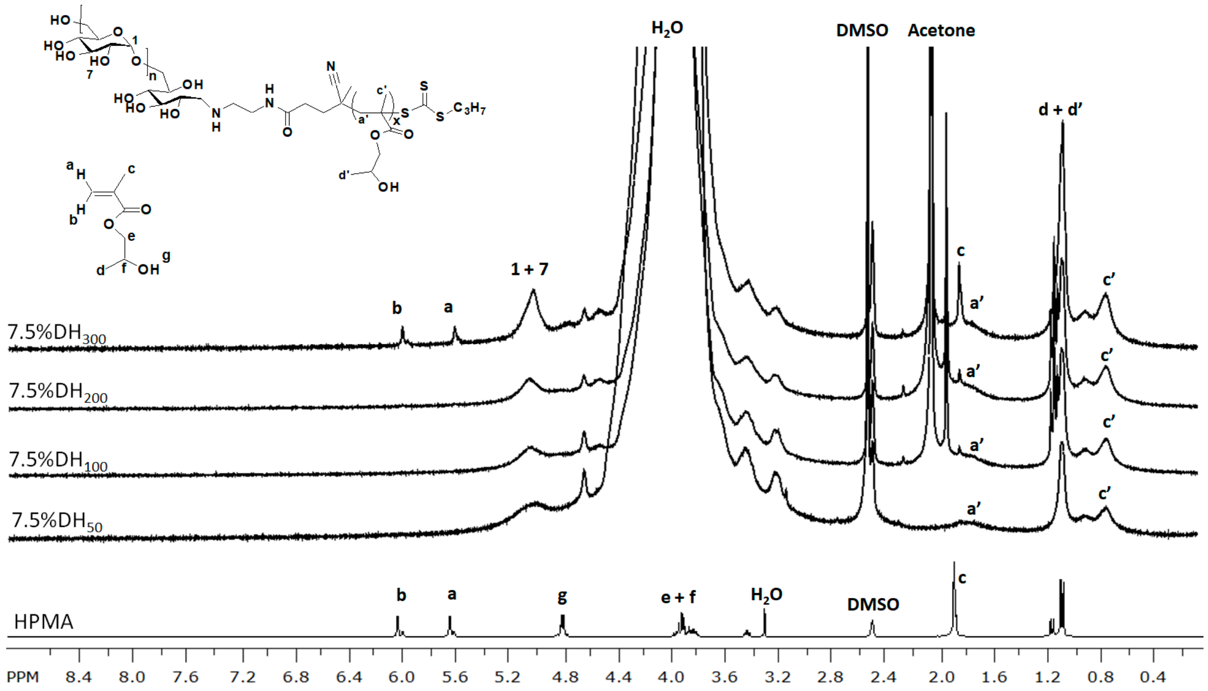

Figure S4. ^{1}H NMR spectra in DMSO-d6 of HPMA and crude suspensions of dextran-*b*-$PHPMA_{X=50-300}$ prepared via photo-RAFT PISA at 12.5% solid concentration.

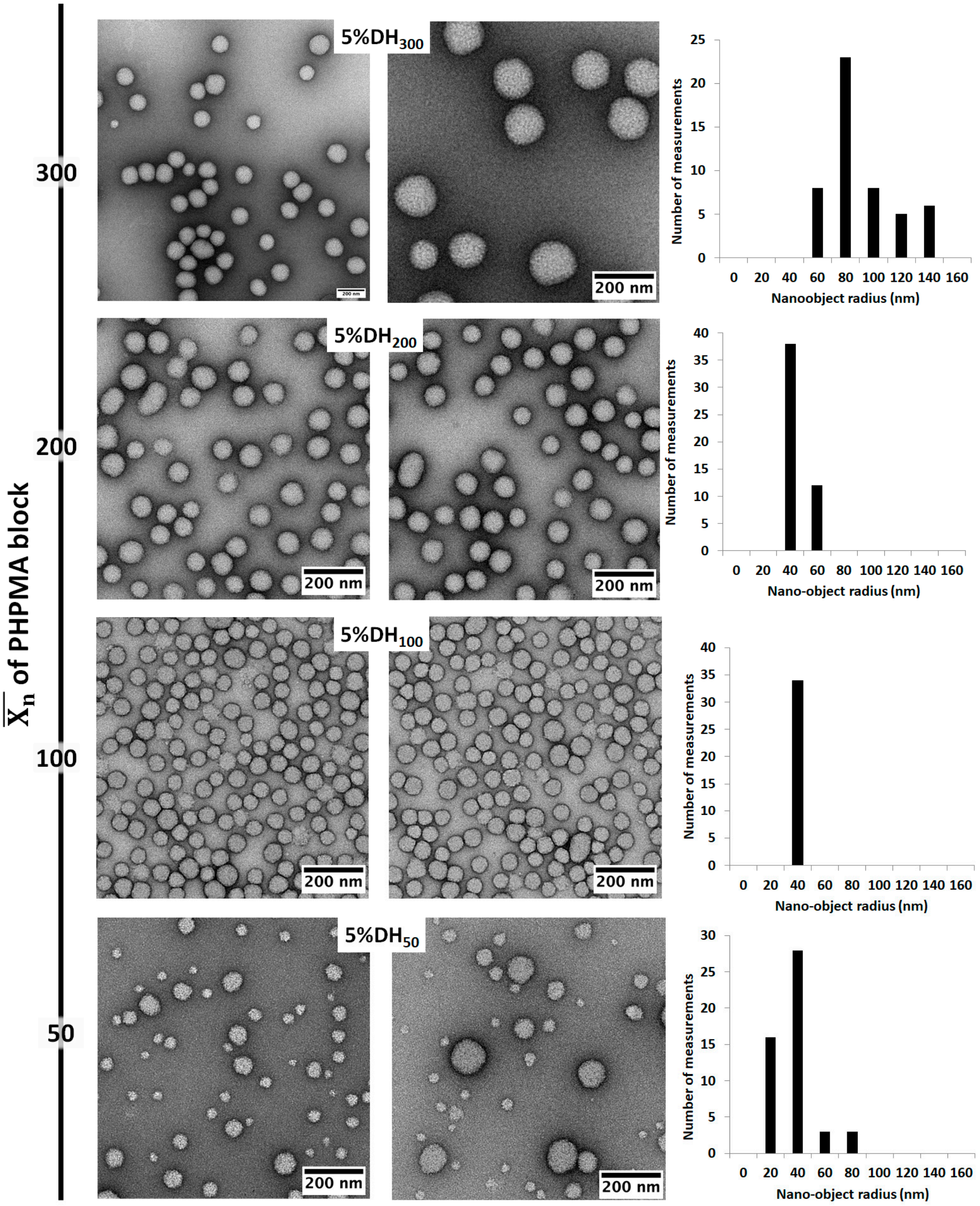

Figure S5. TEM images of dextran-*b*-PHPMA$_{X=50\text{-}300}$ based nano-objects prepared via photo-RAFT PISA at 5% solid concentration. The panels on the right are the statistical analysis of the radii measured for 50 nano-objects using ImageJ software.

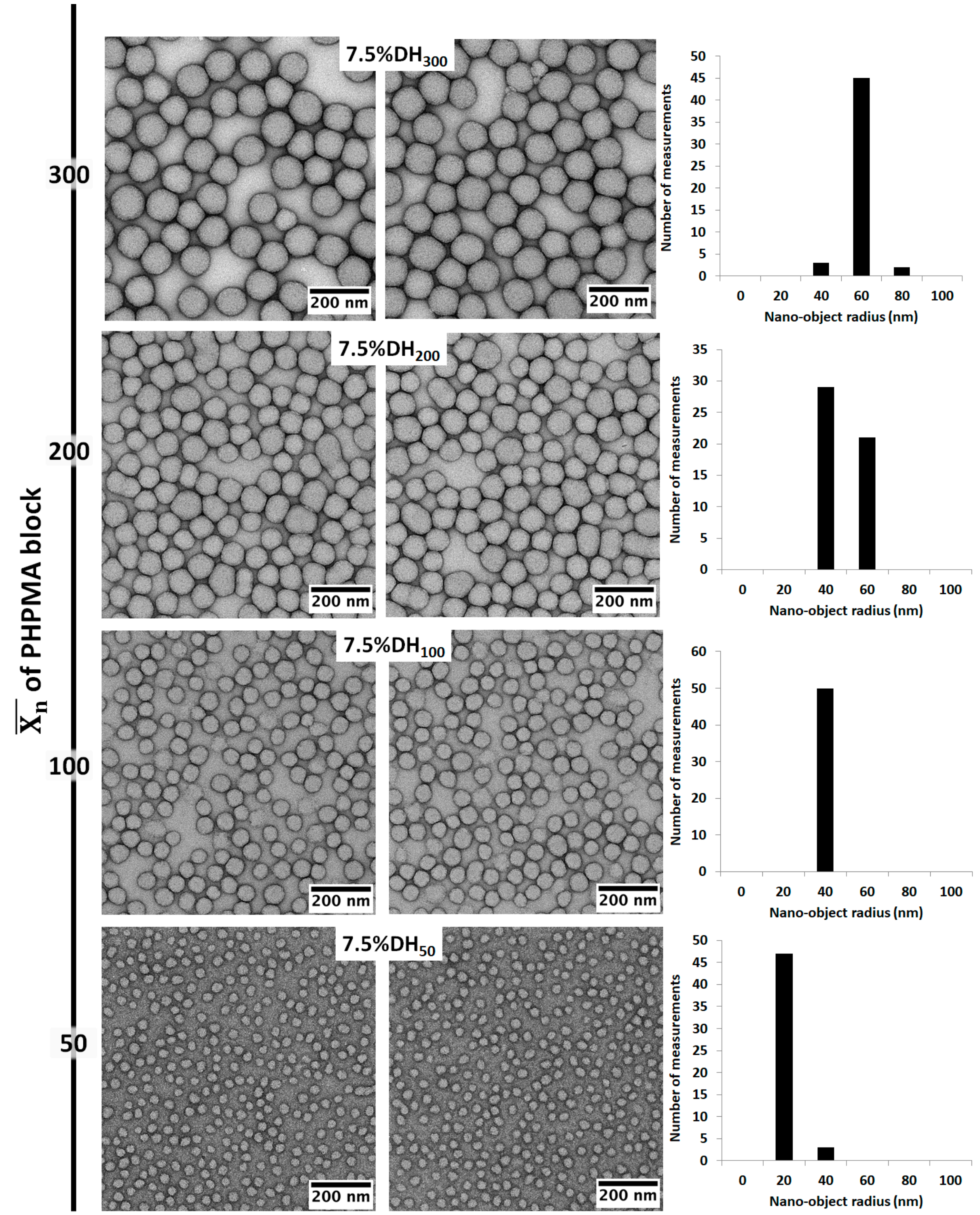

Figure S6. TEM images of dextran-*b*-PHPMA$_{X=50\text{-}300}$ based nano-objects prepared via photo-RAFT PISA at 7.5% solid concentration. The panels on the right are the statistical analysis of the radii measured for 50 nano-objects using ImageJ software.

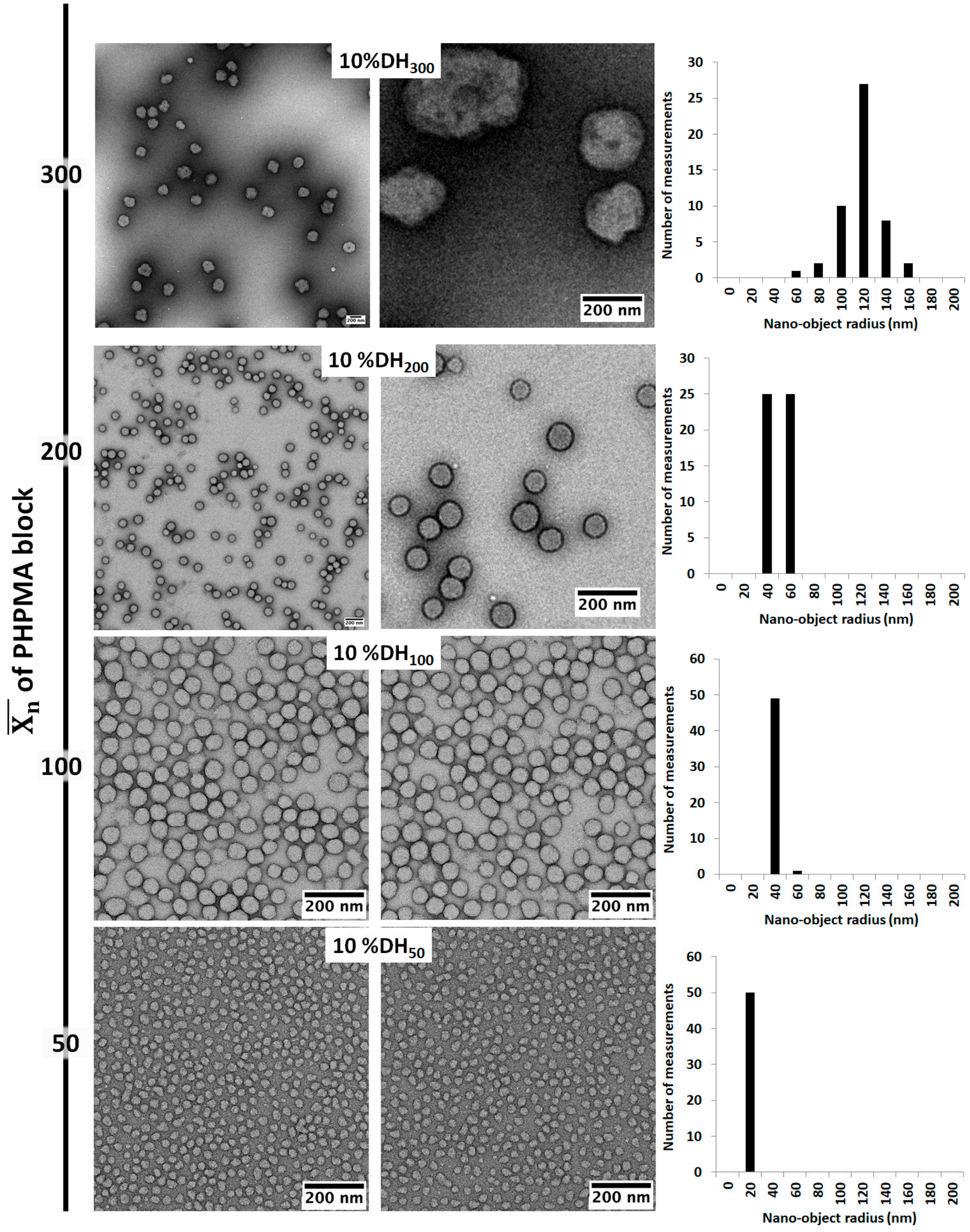

Figure S7. TEM images of dextran-*b*-PHPMA$_{X=50-300}$ based nano-objects prepared via photo-RAFT PISA at 10% solid concentration. The panels on the right are the statistical analysis of the radii measured for 50 nano-objects using ImageJ software.

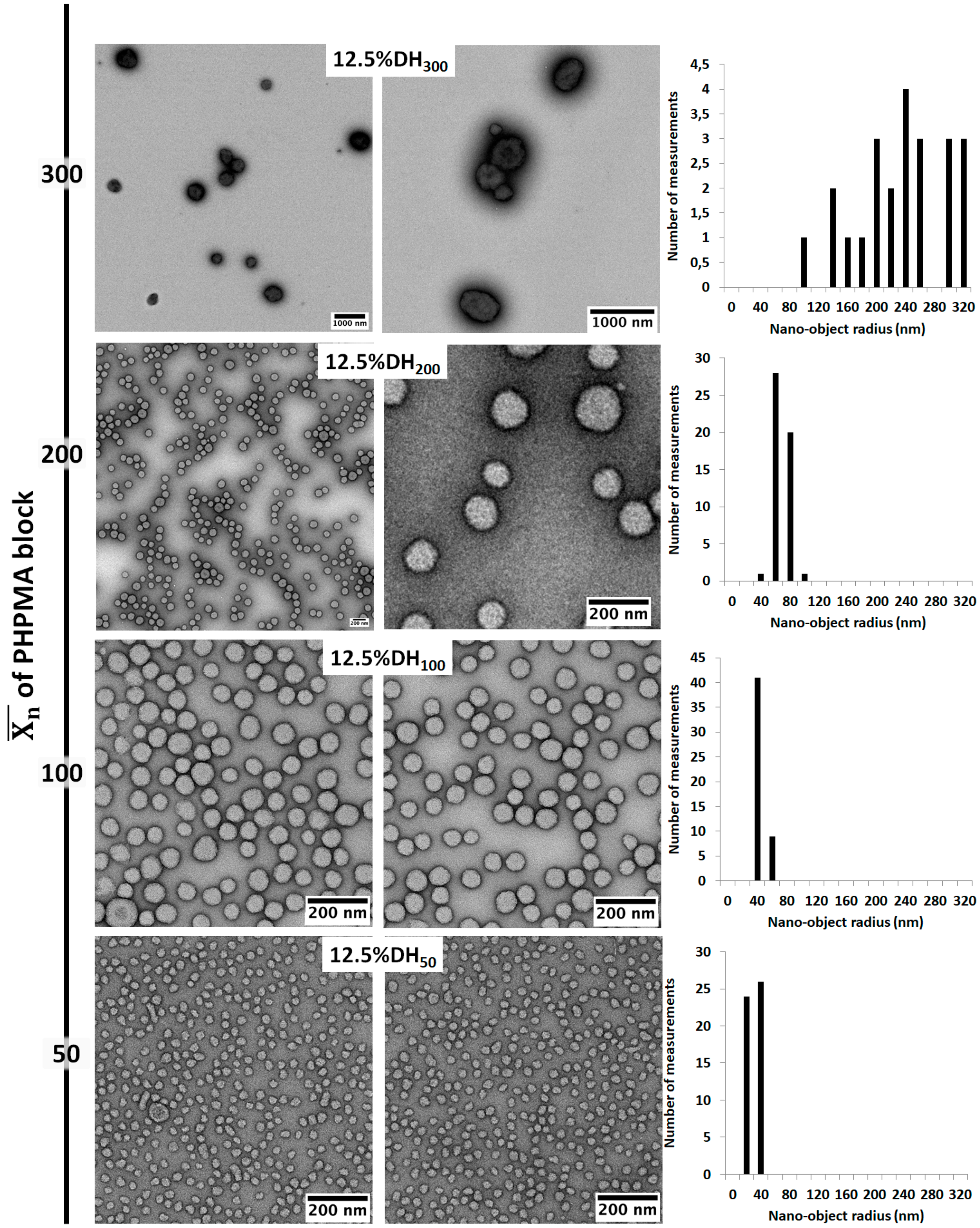

Figure S8. TEM images of dextran-*b*-PHPMA$_{X=50\text{-}300}$ based nano-objects prepared via photo-RAFT PISA at 12.5% solid concentration. The panels on the right are the statistical analysis of the radii measured for 50 nano-objects using ImageJ software.
